# Topiramate for the Treatment of Dually Dependent on Opiates and Cocaine: A Single-center Placebo-controlled Trial

**Published:** 2018-09

**Authors:** Bijan PIRNIA, Ali Akbar SOLEIMANI, Parastoo MALEKANMEHR, Kambiz PIRNIA, Alireza ZAHIRODDIN

**Affiliations:** 1. Dept. of Psychology, Faculty of Humanities, University of Science and Culture, Tehran, Iran; 2. Behavioral Sciences Research Center, Shahid Beheshti University of Medical Sciences, Tehran, Iran; 3. Dept. of Psychology, Faculty of Humanities, Branch of Hamadan, Islamic Azad University, Hamadan, Iran; 4. Technical Assistant in Bijan Center for Substance Abuse Treatment, Tehran, Iran; 5. Dept. of Psychiatry, Behavior Research Center, Shahid Beheshti University of Medical Sciences, Tehran, Iran

**Keywords:** Topiramate (TPM), Anticonvulsants/adverse effects, Methadone maintenance treatment, Cocaine, Opioid

## Abstract

**Background:**

Topiramate facilitates gamma aminobutyric acid (GABA) transference and an ideal candidate for reducing cocaine use in methadone patients. The present study evaluated topiramate in Dual dependence on opiate and cocaine.

**Methods:**

This placebo-controlled study (Clinical Trial Registration Code: TCTR20170201001) conducted during the period 2013–2014, Cocaine-dependent individuals maintained on methadone (n=50) were randomized to receive topiramate or identical placebo capsules. Participants’ dosage ranged between 25–300 mg/day (12 wk) in escalating doses. Methadone Doses started at 30 mg/day (median 100 mg/day; range 20 –140 mg/day). In addition, all subjects received brief behavioral compliance enhancement treatment (BBCET). The data were analyzed by Chi-square Test, generalized estimating equations (GEE) models, linear mixed effects (LME) model and Analysis of covariance (ANCOVA). Primary outcome measures included twelve weekly urine drug screens (cocaine abstinence, detection of benzoylecgonine) and treatment retention. Secondary outcome measures included correlation between cocaine craving with cocaine urine samples and Side effects of depression.

**Results:**

Topiramate was not better than placebo in reducing cocaine use. The secondary outcome showed that Topiramate was better than placebo in reducing cocaine craving. The mean [99% confidence interval (CI)] scores of cocaine craving were 24.31 (18.61–30.01) in experimental group and 21.84 (16.86–26.81) in control group (all *P* > 0.01). Retention and correlation between cocaine craving and cocaine urine samples were not significantly different between the groups. Topiramate usage was not associated with increase in depression symptoms as a side effect (*P*>0.05).

**Conclusion:**

The efficacy of topiramate in cocaine treatment is limited and needs the similar controlled clinical trials and can be used as a complementary intervention.

## Introduction

Opioid dependence is a high prevalence In Iran (1). Methadone maintenance is the first-line treatment of dependency on opioids (2). On the other hand, cocaine usage is common among patients under the treatment of methadone maintainer and is reported between 50% and 76% (3). Simultaneous dependency on cocaine in a large number of methadone users is a troublesome reality that fails the therapeutic consequences (4). Drugs with capability of returning Hemostat glutamate have known as potential remedies of stimulant-dependence (5). Topiramate (TPM) is a kind of anticonvulsant drug suggested by FDA as curing drug of adults’ convulsion and can have significant role in neurological and psychiatric disorders. Topiramate facilitates gamma aminobutyric acid (GABA) transference and prevents Glutamates transference, where this process can reduce the cocaine reinforcing effect and can be used in management of cocaine reliance (6). Topiramate reduces the Presynaptic secretion of glutamate (6). Convulsion can be accounted as a running mechanism in addiction. Therefore, anticonvulsant drugs are counted as suitable candidates for intervention in addiction domain of cocaine reliance (7).

Although the research findings of efficacy of this treatment are contradictive, topiramate is an ideal candidate to increase avoidance in cocaine users (8). In an introductory study over men relied on cocaine, topiramate increased the avoidance period twice the time the placebo did (9). Despite the efficacy of topiramate in reducing cocaine usage, this drug had most application in Cocaine-dependent individuals maintained on methadone. Moreover, this drug has its own usages for other substances, like reporting of hopeful results of decrease in using alcohol (10), gambling craving (11), and Binge eating disorder (12).

In a randomly controlled and closed experiment over cocaine and alcohol users, 20% of those participants that used 300 mg/day topiramate, during the last three weeks showed cocaine avoidance in comparison to those 7 percent who received placebo (13). Significant efficacy of using 300 mg/day in remedy of cocaine reliance was observed. In this study, topiramate users showed 16.6% cocaine avoidance against 5.8% cocaine avoidance of placebo users (14).

We observed the efficacy of this drug in over studies animals. Topiramate in mouse caused reduction of ethanol usage in test of selection of two bottles (Knapp) and reduction of signs of avoidance after remedy of chronic ethanol (15).

Topiramate is an effective and safe treatment for cocaine dependence and is considered as an adjunctive therapy in first four weeks of treatment (16).

On the other hand, some researches gave some evidence of lack of efficiency of topiramate. Topiramate with 200 mg/day did not affect quitting cigarette smoking and preventing alcohol consumption and drug abuse in men addicted to smoking cigarette (17). The evidence do not support using topiramate in improving retention therapy in cocaine usage (18).

Topiramate did not have any significant difference in reducing cocaine usage in methadone-cocaine combined users in comparison to placebo (19). In another study prescription of 200 mg/day topiramate failed in increasing avoidance period of methamphetamine; however, in secondary analysis average level of meth of urine in sample of topiramate group showed significant reduction (20).

In addition, this drug has used for reduction of cocaine craving. Topiramate could reduce avidity intensity and usage period during the out-patience remedy of cocaine users (21). Topiramate could increase period of cocaine avoidance in comparison to placebo group (13).

On the other hand, some studies refer to cognitive side effects caused by topiramate, including slower processing speed, weaker divided attention and an increase in false alarm in recognition memory (22). The most commonly reported side effects due to topiramate; include drowsiness or fatigue, tingling, headaches, digestive disorders and mood disorders such as anxiety and depression (23). Topiramate usage was associated with higher parastzya and difficulty in attention compared to the control group (24). Selecting the desired range in topiramate dose can minimize side effects (25).

Considering the conflicting results about the effectiveness of topiramate in cocaine dependency treatment, also, existing evidence about physical and psychological side effects of topiramate usage, this study aimed to examine the effectiveness of topiramate on amount of cocaine usage, reduction of craving, the relationship between craving and the amount of usage and side effects of topiramate.

## Materials and Methods

This study was carried out on all of the Cocaine-dependent individuals maintained on methadone in Bijan Center for Substance Abuse Treatment. From this community, 54 persons were selected Respondent-driven sampling (25) and based on the information obtained from previous studies. The data of this study were gathered from Dec 15, 2013, until Nov 20, 2014. In during the 4 weeks before randomization, Screening were completed. Physical examination, tuberculin skin test, chest X-ray, Urine toxicology and electrolytes completed during screening. Tuberculin skin test was conducted to evaluate the possible association with diabetes. Tuberculosis and diabetes mellitus have synergetic relationship and suffering from diabetes was considered as one of the inclusion criteria of the research (item number 7). Chest X-ray study to evaluate Dyspanya as one of the side effects of topiramate was done in the evaluations of the screening stage. Urinalysis or urine toxicology refers to the evaluation of metabolites of cocaine with the threshold of 300 ng/mL included as a criterion to matching the subjects in doses of use at screening. Sometimes after the research process (between third to ninth week), four people were removed from the experiment for many reasons like absence in remedial sessions and positive urine test. Fifty participants were randomly assigned to two groups (25 persons each) of experimental and placebo. Inclusion criteria were: 1) between 18 – 55 yr old; 2) eligible for cocaine-dependent individuals maintained on methadone. The Exclusion criteria were: 1) report of positive urine test during the remedial period; 2) sulfonamide or Hypersensitivity to topiramate; 3) nephrolithiasis or renal impairment, kidney failure history, serious mental disorders and court-mandated drug abuse treatment; 4) using carbonic anhydrase inhibitors; 5) glaucoma, family history of glaucoma, intraocular hypertension or one-sided blindness; 6) seizure disorder or use of antiepileptic medications or current benzodiazepine dependence; 7) diabetes, respiratory insufficiency, or other chronic risk factor for acidosis.

All the topiramate capsules together with monohydrate lactose powder were provided less than sixty days before usage. Lactose was combined with 5 PPM denatonium benzoate to assimilate the bitter taste of all the capsules (similar to placebo). Both types of capsules were opaque and were of the same size of 550 mg. The 200 mg/day base dose topiramate was used in this study with regard to rare side effects of dosage of lower than 400 mg/day reported of this drug in clinical studies. Doses were scaled from small doses and the starting dose was 25 mg/day until maximum of 300 mg/day pills. The daily usage was divided into two dosages of morning and afternoon. The urine test band was randomly used to study cocaine metabolite in first four weeks in form of two weekly evaluations and after that once every fifteen days. The validity of the samples controlled by studying urine temperature, Creatinine and PH and threshold of 300 ng/ml was used for the urine analysis.

The urine specimens were collected every Monday and Friday from 10 a.m. to 6 PPM and were analyzed by enzyme-multiplied immunoassay technique (EMIT) System for benzoylecgonine equivalents (cocaine) with cutoff concentrations for Positive set at 300 ng/ml for 24 to 60 h after last use. Test sensitivity is 99% and specificity is 98%. After tests inspection, participants were motivated to continue taking part in research through email. To ensure the commitment of subjects to usage avoidance, urine test was taken from control group too. Furthermore self-reporting index of cocaine craving in twelve weekly and depression was evaluated in pretest and posttest. Moreover, participant interviews were audio-recorded and transcribed.

Methadone (methadone 5 mg/mL) was administered daily by nursing staff. Doses started at 30 mg/day and inducted over three weeks to a maintenance dose (median 100 mg/day; range 20–140 mg/day). Take home doses were provided on holidays and for rare emergencies.

Once per week during the treatment phase, all subjects received brief behavioral compliance enhancement treatment (BBCET) to promoting compliance with the study medication.

### Sample size, Randomization, and blinding

The sample size was calculated to be 50 subjects. In this study, with regard to one-way direction of Test and assumption of Z=1.645, d=0.2, *α* =0.05 and also power of test 1-*β*=0.84 and using the following formula:

$$\text{n}=\text{z}+(1-B)/{\text{d}}^{2}={\left(1.645+0.84\right)}^{2}/.25=24.7\approx 25$$

Where:

n: = (n) q
n: = 25 × 2 = 50

In the first step, 54 participants registered for participating in the study. Sometime after the beginning of the study (between third week and ninth week) four persons were removed from the study because of many reasons like absence from remedial sessions and report of positive urine test. Fifty participants were randomized tointowo groups (25 persons in each group) through IBM SPSS Statistics ver. 20 (IBM Corp., Armonk, NY, USA) (random number generation).

### Statistical analysis

We used generalized estimating equation (EEG) model to compare the groups on weekly cocaine use based on UCT and craving with time variable (Diggle). To estimate, the correlation coefficient between two variables with repeated observations, using linear mixed effects (LME) model. After the passage of twelve remedial sessions, all of the participants of the study were evaluated for the second time but this time in form of a posttest through Depression questionnaire by Analysis of covariance (ANCOVA).

### Trial Registration

The trial was registered at the Thai Clinical Trials Registry (http://www.clinicaltrials.in.th) with the TCTR ID: TCTR20170201001.

Structured clinical interview, demographic researcher-made questionnaire, Cocaine Craving Questionnaire, Beck Depression Inventory, and Urine sample test were used.

***Clinical structured interview for disorders (SCID):*** It is a clinical interview used for distinguishing axis-one disorders based on DSM-IV. In previous studies the final coefficient for measures of SCID was reported 0.60 (26). The identification agreement of this instrument in Persian language was useful for the most of special and general determinations with reliability of higher than 0.60. Kappa coefficient for all of current determinations and determination of lifetime were 0.52 and 0.55 respectively (27).

***Cocaine Craving Questionnaire:*** was designed by (28). The abridged version of it includes 10 statements that its psychometric features were investigated by (29) on a sample including 247 cocaine users. The correlation of this index with Beck’s depression index 0.39, anxiety index 0.35 and with recent use of drugs 0.26 was reported. The correlation of abridged form with original form of questionnaire was estimated 0.85 and the internal reliability of this measure according to Corn Bache alpha was estimated 0.90.

***Urine Benzoylecgonine Tests (UBT):*** in form of Cocaine test representing addiction were taken randomly in a period of four weeks in form of two times per week and after that once every fifteen days.

***Beck Depression Inventory (BDI):*** is vastly used in evaluating the intensity of depression and is of high reliability and efficacy. This tool is a self-reporting tool that has 21 items. Each item is graded in a range of 0 to 3 and the mean shows the global score of depression and the highest score shows the highest score for depression.

## Results

**Fig. 1:**
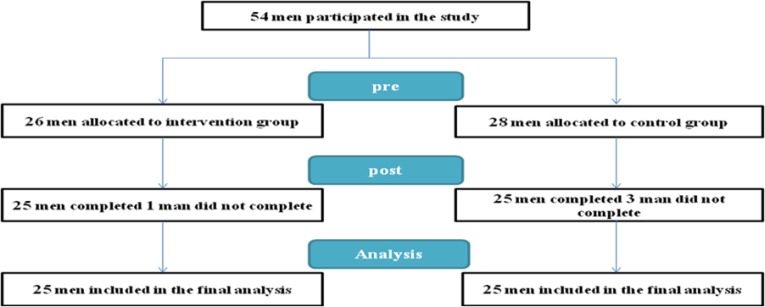
Trial diagram

In the first step, 54 participants registered for participating in the study. Sometime after the beginning of the study (between third week and ninth week), two persons were removed from the study because of many reasons like absence from remedial sessions and reports of positive urine test, 2 participants were discharged for medical reasons: 1 paranoid psychosis; 1 pneumonia. Fifty Participants were randomized by a Microsoft Excel macro to two groups (25 persons in each group) of experimental and control one (Fig. 1). Table 1 shows the demographic state of the participants of the study.

**Table 1:** Demographic status of the participants of the study

***Variable***	***TPM n (%)***	***Placebo n (%)***
Education		
Lower than Diploma	9(16)	7(28)
Higher than Diploma	16(64)	18(72)
Age(yr)		
18–25	17(68)*	15(60)*
Older than 25	8(32)	10(40)
Employment		
employed	11(44)	12(48)*
unemployed	14(56)	13(52)
Monthly income		
Less than 200 dollars	14(56)	13(52)
More than 200 dollars	11(44)	12(48)

* *P*<0.01

Most of the subjects have the educational level higher than Diploma. Concerning age, most of the participants have the age index of lower than 25 yr. Most of the participants in both groups have the income of less than $200 per month.

Data analysis using Chi-square test showed that the distribution of participants in case of demographic variables shows significant difference in two indices including age and Education level (all *P*<0.01).

There is no significant difference between two groups in case of the amount of usage during 12 wk. In case of usage craving, weekly comparison indicates significant difference. Data analysis using linear mixed effects (LME) test shows that there is not a significant relationship between two variables of the cocaine craving and cocaine urine samples. Data analysis using analysis of covariance showed that topiramate was not associated with increase in depression symptoms as a side effect (*P*>0/05) (Table 2).

**Table 2:** Multivariate GEE analysis of predictors for ongoing drug use during 3 month TPM in Urine Cocaine test + Cocaine craving

***Variable***	***Topiramate N=25***	***Placebo N=25***	***Pairwise Comparisons***
Urine Cocaine Test	11.24 (1.82)	11.08 (2.13)	NS
Cocaine craving	24.31 (2.21)	21.84 (1.93)	*P* < 0.01

## Discussion

The primary result of the present study about the lack of effectiveness of topiramate treatment in reducing cocaine use based on urine test in the Cocaine-dependent individuals maintained on methadone. This finding it is in according to the current evidence base for topiramate treatment. For example, the topiramate was not better than placebo in promoting cocaine or alcohol abstinence (21).

Similarly, the results of a study (20) on a sample of 140 methamphetamine users showed topiramate reduced the weekly mean of urine test of methamphetamine and also the severity of dependency in participants of the study between 6 to 12 wk (similar to the present study) but topiramate failed to increase abstinence from methamphetamine.

This result is in contrast to studies in the treatment of cocaine dependence compared to the placebo (30) that found significant effect of topiramate (300 mg/day). The study by Johnson and the present study are different in two components. The first one is the sample size and the other one is that in study by Johnson the basic dose of 50 mg/day was used but in the present study the basic dose of 25 mg was used. Although, two studies showed topiramate could decrease craving for use independent users (30). Nevertheless, topiramate could not increase the duration of abstinence in participants, although the participants with negative urine test showed longer abstinence duration. Although the structure of the present study has some similarities with the mentioned research the structural nature of cocaine and its difference in case of absorption with methamphetamine makes the comparison difficult. Although there is difference in basic dose in the present study that as origin of difference in the efficacy. Moreover, in contrast to our findings, topiramate decreased the days of usage in individuals’ dependent to cocaine in three studies, although the role of individual differences in efficacy of the treatment is mentioned (31). Topiramate, in spite of efficacy and not being dangerous, just was effective on crack cocaine users in the first four weeks of the treatment (16). In the first four weeks, topiramate could decrease the usage frequency. In a study inconsistent with our results, the effectiveness of topiramate were reported on increasing negative urine test of methamphetamine in a sample of Iranian users in the sixth week, significant (32).

Consistent with our findings, in meta-analysis study (18) conducted with the aim of examining the effectiveness of topiramate in five types of research, four comparative studies with placebo group and one study with non-pharmacological intervention, the results showed topiramate did not show a significant difference with placebo in cocaine usage abstinence. In this regard, limited effectiveness of topiramate was reported in crack cocaine users (33). In contrast to previous studies, fixed dose of 200 mg was used, a component that claims to challenge the efficacy of topiramate in high dose. In regard to our results, a meta-analysis (7) also showed the effectiveness of topiramate lacks clinical evidence and is not useful for the first line of treatment and it deserves to be used in terms of a complementary treatment.

Findings of the present study are consistent with the results of study (16) that showed topiramate is an effective and safe treatment for cocaine dependency. The results of the present study are in contrast with the finding (18) that conducted a meta-analysis of clinical trials and reported that the effectiveness of topiramate in treating cocaine dependency was low and not significant.

Moreover, secondary outcome of the present study about the effectiveness of topiramate on reducing usage craving is consistent with the results of other studies (30). Topiramate showed significant difference in increasing craving for usage in one of the five studies (31). Finally, in terms of a pilot on Iranian samples, 10-week prescription of topiramate in terms of ascending dose on decreasing craving for usage in methamphetamine users was reported effective (32).

The results of Beck’s Depression test in examining the side effects due to topiramate usage showed that in the present study, topiramate usage was not associated with increase in depression symptoms. The results of the present study are in contrast with findings of meta-analysis (23) in which prescribing topiramate has been reported with symptoms of anxiety and depression. In explaining the findings of the present study, this reality can be referred that selecting desirable range in topiramate dose can minimize the side effects (24). Overall, efficacy of topiramate in cocaine treatment is limited and needs the similar controlled clinical trials and can be used as a complementary intervention.

The most important our limitations were as follows: 1) the cross-sectional nature of the study limits the overall conclusion and comprehensive forecast; and 2) using a self-report assessment in sensitive subjects often creates a favorable social image and thus, self-reporting is associated with possible bias.

## Conclusion

Briefly, adding topiramate to treatment with methadone maintenance in dual methadone-cocaine users, have not created significant difference in reducing cocaine usage. However, topiramate treatment could reduce the amount of craving in participants of experimental group, although it did not have that much effect on durability of the treatment and a significant difference was not seen between the amount of usage and usage craving. Also, prescription of topiramate was not associated with psychological side effects such as depression symptoms. The findings can be important in case of application in the process of preventing the slip and the continuity of usage avoidance in dual users. It is recommended to conduct a complementary research on wider samples and including female subjects.

## Ethical considerations

Ethical issues (Including plagiarism, informed consent, misconduct, data fabrication and/or falsification, double publication and/or submission, redundancy, etc.) have been completely observed by the authors.
